# Molecular features of glioblastomas in long-term survivors compared to short-term survivors—a matched-pair analysis

**DOI:** 10.1186/s13014-022-01984-w

**Published:** 2022-01-24

**Authors:** Vivien N. Sommerlath, Daniel Buergy, Nima Etminan, Stefanie Brehmer, David Reuss, Gustavo R. Sarria, Marie-Christin Guiot, Daniel Hänggi, Frederik Wenz, Kevin Petrecca, Frank A. Giordano

**Affiliations:** 1 Department of Radiation Oncology, University Medical Center Mannheim, University of Heidelberg, Heidelberg, Germany; 2 Department of Neurosurgery, University Medical Center Mannheim, University of Heidelberg, Heidelberg, Germany; 3 Department of Neuropathology, Institute of Pathology, University of Heidelberg, Heidelberg, Germany; 4 Department of Radiation Oncology, University Hospital Bonn, University of Bonn, Venusberg-Campus 1, 53127 Bonn, Germany; 5 Department of Pathology, Montreal Neurological Institute, Montreal, Canada; 6 Department of Neurosurgery, Heinrich-Heine-Universität, Düsseldorf, Germany; 7 Freiburg Medical Center, Freiburg, Germany; 8 Department of Neurosurgery, Montreal Neurological Institute, Montreal, Canada

**Keywords:** Glioblastoma, GBM, Prognostic factors, GFAP, MGMT

## Abstract

**Background:**

Although glioblastoma (GB) is associated with a devastating prognosis, a small proportion of patients achieve long-term survival rates. We herein present a matched-pair analysis of molecular factors found in long- and short-term survivors (LTS, STS).

**Methods:**

We performed a cross-institutional analysis of 262 patient records and matched a group of 91 LTS (≥ 3 years) with two groups of STS (STS-1, n = 91; STS-2, n = 80). Matching was performed according to age, Karnofsky Performance Status, initial therapy and adjuvant therapy. Molecular factors were compared between LTS (total of 91 patients) *v.* STS-1, and LTS (subgroup of 80 patients) *v.* STS-2. We included glial fibrillary acidic protein (GFAP), O6-methylguanine-DNA methyltransferase (MGMT) promoter methylation, isocitrate dehydrogenase 1 (IDH-1); furthermore, the proliferation index was analyzed (Ki-67/MIB-1).

**Results:**

IDH-1 and decreased Ki-67 were numerically associated with LTS but the difference was only significant compared to STS-1 (n.s. *v.* STS-2). LTS was associated with MGMT promoter hypermethylation (*p* = 0.013 and *p* = 0.022) and GFAP expression (*p* < 0.001 and *p* = 0.001). Positivity for both factors combined compared to negativity for one factor occurred more often in the LTS group (*p* = 0.002 and *p* = 0.006); negativity for both factors combined did not occur in the LTS group.

**Conclusion:**

In this retrospective analysis, GFAP expression and MGMT promoter methylation were associated with LTS. Given the hypothesis-generating nature of our study, these observations should be confirmed in prospective clinical trials.

**Supplementary Information:**

The online version contains supplementary material available at 10.1186/s13014-022-01984-w.

## Introduction

Glioblastoma (GB) represents the most common primary malignancy of the brain [1] and it is known for having one of the worst survival rates among all cancers [2, 3]. Nevertheless, a small proportion of patients diagnosed with GB achieves long-term survival [4]; available data indicate that 2 to 5% of patients survive 3 years or more, thus classified as long-term survivors (LTS) [5, 6].

The 2016 World Health Organization (WHO) classification classifies gliomas not only based on histopathologic features but also on molecular [7] parameters. These include 1p/19q codeletion for oligodendroglial tumors but not for anaplastic astrocytoma or GB; furthermore, astrocytoma, including GB are classified as isocitrate dehydrogenase (IDH)-mutant or wildtype (or NOS in inconclusive cases). The most common *IDH* mutation which accounts for about 90% of all identified *IDH* mutations is *IDH1* R132H [8]. Another relevant molecular feature includes O6-methylguanine-DNA methyltransferase (*MGMT*) promoter methylation. MGMT is a DNA repair enzyme. In case of *MGMT* gene promoter methylation, the gene is silenced thereby impeding DNA damage repair caused by alkylating agent chemotherapy leading to an increased effectiveness of chemotherapy [9, 10]. The prognostic value of MGMT promoter methylation for progression-free survival (PFS) and overall survival (OS) has been suggested by multiple studies and meta-analysis (e. g. Zhao et al. [11]). Tumor growth fractions using the Ki-67 index in GB have been studied using different antibodies, including Molecular Immunology Borstel-1 (MIB-1) which has been used to label Ki-67 in glioma samples by multiple groups [12–14]; however, the prognostic relevance of such approaches remains uncertain with conflicting results in multiple studies [12–15]. Finally, glial fibrillary acidic protein (GFAP) has been proposed as a diagnostic marker for GB [16, 17]. Loss of GFAP in astrocytoma has been associated with high-grade tumors [18, 19] and cellular loss is associated with faster tumor growth [20]. However, rodent models indicated that loss of GFAP does not contribute directly to tumor progression [21] and its prognostic relevance is not well-established.

We conducted a bi-institutional matched-pair analysis to evaluate which of the aforementioned histopathologic or molecular parameters might be associated with OS in patients with GB.

## Patients and methods

In this cross-institutional matched-pair analysis, patients from the Neurological Institute Montreal, McGill University, Canada and from the department of Radiation Oncology, University Medical Center Mannheim, Germany were included. Local institutional review board (IRB) approval was obtained for this study (2013-832R-MA). We identified a cohort of LTS with an OS of 36 months or longer and matched them to two cohorts of STS (STS-1 and STS-2). Matching of patients was performed according to age, Karnofsky Performance Status (KPS), initial therapy and adjuvant therapy. Extent of surgery was estimated using surgical reports. Most patients (91.6%) were treated between 2006 and 2018 with 8.4% of patients diagnosed between 2001 and 2005.

Histopathological parameters which were analyzed in this study included Ki-67 which was evaluated using the MIB-1 antibody or a Ki-67 antibody as part of the clinical routine workup. Furthermore, GFAP expression in available tumor samples was classified as positive, vs. negative. Tumor samples in which GFAP expression was partially lost were counted as negative. MGMT promoter expression was classified as methylated or unmethylated. For this study, staining for *IDH1* R132h mutations was performed and either classified as mutated or wildtype; sequencing results for other *IDH* mutations in case of *IDH1* R132h wildtype were not analyzed in this study.

### Statistical analysis

We applied the McNemar test for dichotomous variables in the matched-pair comparison; in case of < 25 discordant cells, the binomial exact test was used. Non-parametric continuous variables in the matched-pair comparison were analyzed using the Wilcoxon test. All variables which were significant in the first comparison (LTS v. STS-1) were reanalyzed in a confirmation analysis using the 2nd STS-2 match which was used as a confirmation set for STS but no second dataset was available for the LTS group. Statistical significance threshold was set at 0.05 (*p* < 0.05); furthermore, both STS-1 and STS-2 were required to be significantly different compared to the LTS; we did not additionally account for multiple testing. Finally, all significant variables were analyzed in a Kaplan–Meier model to estimate the relevance of the clinical benefit in months. Statistical analyzes were performed using R [22], a language and environment for statistical computing or SPSS (IBM, Armonk, NY, USA).

## Results

The primary cohort consists of 91 LTS, they were matched to an STS cohort (*n* = 91); furthermore, we identified a second cohort of STS (n = 80) to match them with 80 patients of the original LTS group.

### Clinical parameters

Median age for LTS, STS-1, and STS-2 were 54, 56, and 55.5 years, respectively. At least partial surgical resection as primary treatment was performed in 94.5%, 94.5%, and 95% while biopsy only was performed in 5.5%, 5.5%, and 5% of patients. Primary tumor locations were left-sided in 49.5%, 46.2%, and 53.8% and both-sided in 3.3%, 6.6%, and 3.8% of patients. All clinical parameters of LTS, STS-1, and STS-2 and the 80-patient subgroup of LTS which were matched to STS-2 are detailed in Table 1.

**Table 1:** Clinical features of analyzed patients

Clinical features	Specification	All	LTS (*n* = 91)	STS-1 (*n* = 91)	LTS (subgroup, *n* = 80)	STS-2 (*n* = 80)
KPS	Median (range)	80 (30–100)	80 (30–100)	80 (40–100)	70 (30–100)	80 (30–100)
Age	Median (range)	55 (11–84)	54 (22–77)	56 (11–84)	56 (22–77)	55.5 (15–77)
Gender (%)	Female	42.7	45.1	52.7	42.5	28.8
	Male	57.3	54.9	47.3	57.5	71.3
Surgery/biopsy (%)	Surgery	94.7	94.5	94.5	95.0	95.0
	Biopsy	5.3	5.5	5.5	5.0	5.0
Dose	Median (range)	60 (7–68)	60 (37.5–60)	60 (16–68)	60 (37.5–60)	60 (7–62)
Multifocal* (%)	Yes	65.1	50.0	81.3	50.0	66.7
	No	34.9	50.0	18.8	50.0	33.3

* Results refer to patients in whom CT scans were available

In LTS, STS-1, and STS-2 89%, 95.6% and 93.8% of patients had received radiochemotherapy with a median radiotherapy dose of 60 Gy (range: 7–68 Gy) in all groups (statistical average: 58.9 Gy, 58.6 Gy, and 57.8 Gy for LTS, STS-1, and STS-2, respectively). 8.8%, 4.4% and 6.3% of LTS, STS-1 and STS-2 received radiotherapy alone. After completion of radiotherapy (± concurrent chemotherapy) adjuvant chemotherapy was given in 89.0%, 79.1% and 82.5% in LTS, STS-1 and STS-2 (mostly including TMZ 82.4%, 70.3% and 76.3% of the cases).

### Survival, histopathological and molecular markers

**Fig. 1:**
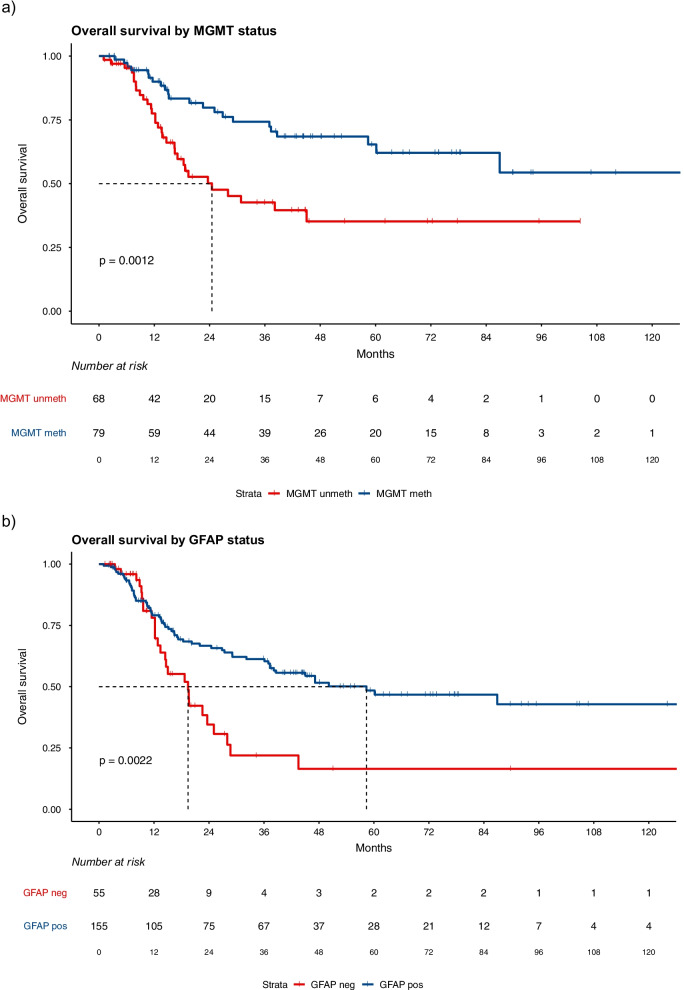
**a** Overall survival function comparing MGMT promoter methylated tumors with unmethylated tumors. Hypermethylation of the MGMT promoter region was associated with an increase in OS (*p* = 0.0012). Survival in both groups is longer compared to an unselected group of GB patients due to the artificially high number of long-term survivors (55 patients; 37.4%). All patients with available MGMT data are Included in this graph (n = 147). **b** Overall survival function comparing tumors which were at least partially negative for GFAP with tumors in which samples stained positive for GFAP. The difference between the groups was significant (*p* = 0.0022). As in **a**, survival in both groups is longer compared to an unselected group of GB patients due to the artificially high number of long-term survivors (72 patients; 34.3%). All patients with available GFAP data are included in this graph (n = 210)

**Fig. 2:**
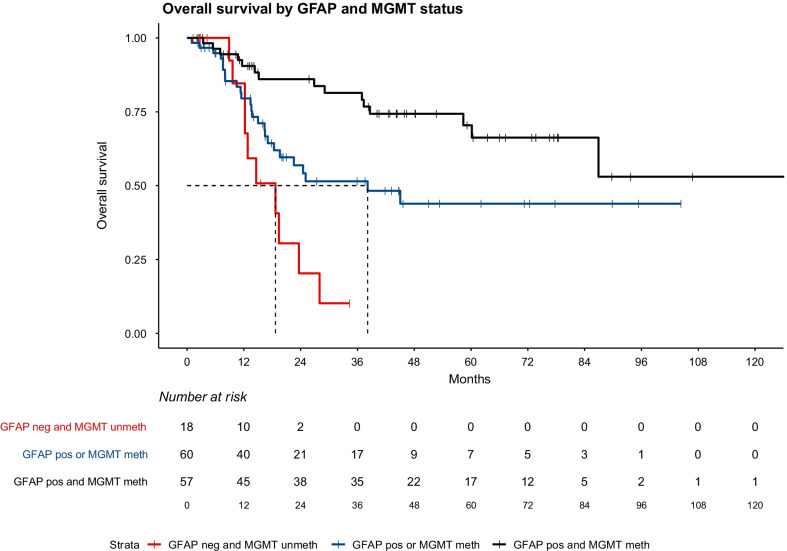
This figure shows the OS in the overall population (LTS, STS-1, STS-2) by MGMT and GFAP status; the black curve represents patients which are positive for GFAP and also have a hypermethylated *MGMT* promoter region. The blue curve represents patients with mixed expression; i.e., either a hypermethylated MGMT promoter region combined with an at least partially negative GFAP expression in the tumor and vice versa. The red curve shows OS outcomes for patients who had an at least partially negative GFAP staining in the tumor and who had an unmethylated *MGMT* promoter region. Differences between the double-positive patients compared to mixed patients was significant (*p* = 0.01) and the difference between mixed patients and double-negative patients was also significant (*p* = 0.038)

After a median follow-up of 16 months (range 1–156) (53.3, 9.6, and 11.8 months for LTS, STS-1, and STS-2, respectively), the median OS in the full LTS cohort and in the partial LTS cohort which included the 80 patients matched to STS-2 were not reached (lower bound of the 95%-confidence interval also not reached). Median OS in the first matched STS cohort of 91 patients (STS-1) was 13.4 months (12.2–20.2). In the 2nd matched STS cohort (STS-2) for which 80 patients could be identified, median OS was 16.4 months (13.6–19.4). Kaplan–Meier curves for both STS groups and for the full and the partial LTS group are shown in Additional file 1: Figures S1a-b. Growth fraction (Ki-67) of tumors was available in 208 samples (79.4%) out of which 126 had been processed using the MIB-1 antibody. We identified a significantly lower Ki-67 index in the LTS group when compared to STS-1 (median: 18% (range 5–60%) *v.* 30% (range 8–90%); *p* < 0.001); however, the difference was only a trend in the control comparison out of 80 LTS patients with the STS-2 group (20% *v.* 25%; *p* = 0.085). Data on Ki-67 were unavailable in 54 patients. *IDH1* R132h staining was done in 136 samples; out of patients with available data, mutations were present in 15.4% of patients in the LTS group which was significantly higher compared to the STS-1 group (7.3%; *p* = 0.031) but not compared to the 2nd control match (STS-2; 11.9%; *p* = 0.125). MGMT methylation was unknown in 43.9% of patients; in patients with available data positive methylation was observed more frequently in the LTS group (70.9%) compared to STS-1 (49%; *p* = 0.013) and STS-2 (37.2%; *p* = 0.022). GFAP expression was only categorized as positive if expression was detected in all analyzed slides; if GFAP expression was lost in a single fraction of tumor cells the tumor was classified to be overall negative. GFAP expression data were available in 80.2% of patients; in the LTS group, the frequency of GFAP-positive tumors was higher (94.4%) compared to STS-1 (61.6%; *p* < 0.001), and STS-2 (64.6%; *p* = 0.001), both of which included more tumors which were at least partially negative for GFAP (STS-1: 38.4%; STS-2: 35.4%). All molecular features for LTS, STS-1, and STS-2 groups are shown in Table 2. Both MGMT and GFAP were additionally analyzed using Kaplan–Meier models for the overall population (LTS, STS-1, and STS-2). These models are shown in Fig. 1a, b, both showed a significant association of MGMT and GFAP expression with OS (*p* = 0.0012 for MGMT and *p* = 0.0022 for GFAP). Expression of *GFAP* was not associated with *MGMT* hypermethylation in the overall study cohort and both factors were analyzed combined, negativity for both factors was associated with a worse outcome compared to positivity for one (*p* = 0.038); positivity for both factors was associated with better outcomes compared to positivity for one (*p* = 0.01; see Fig. 2). Positivity for both factors (GFAP positive and MGMT methylated) also occurred more frequently in the group of LTS (66% of patients with available data on both) compared to both STS groups (28.6% and *p* = 0.002 for STS-1; 25% and *p* = 0.006 for STS-2). There were no patients who were negative for both factors in the LTS group while STS-1 and STS-2 included 19% and 25% out of those with available data, respectively.

**Table 2:** Molecular features of analyzed patients

Molecular features	Specification	All	LTS (n = 91)	STS-1 (n = 91)	LTS (subgroup, n = 80)	STS-2 (n = 80)
GFAP* (%)	Positive	73.8	94.4	61.6	93.5	64.6
	Partially negative	25.2	5.6	37.0	6.5	33.8
	Negative	1.0	0	1.4	0	1.5
MGMT* (%)	Methylated	53.7	70.9	49.0	71.1	37.2
	Unmethylated	46.3	29.1	51.0	28.9	62.8
IDH1R132H* (%)	Positive	11.0	15.4	7.3	16.7	11.9
	Negative	89.0	84.6	92.7	83.3	88.1
MIB1	Median (range)	20 (3–90)	18 (5–60)	25 (8–50)	18 (5–60)	25 (3–90)
Ki67	Median (range)	25 (8–90)	20 (8–60)	30 (10–90)	25 (10–60)	25 (10–80)
Ki67/MIB1	Median (range)	25 (3–90)	18 (5–60)	30 (8–90)	20 (5–60)	25 (3–90)
GFAP/MGMT* (%)	GFAP+/MGMT+	42	66.0	28.6	65.9	25.0
	GFAP+/MGMT−	34	30.2	35.7	29.5	37.5
	GFAP−/MGMT+	10	3.8	16.7	4.5	12.5
	GFAP−/MGMT−	13	0	19.0	0	25.0

*Results refer to patients in whom tissue was available

## Discussion

The aim of our study was to evaluate prognostic molecular factors in a large sample of GB patients who achieved a long-term survival. Several clinical and therapeutic factors have been reported to be associated with OS in patients with GB. Specifically, resection compared to biopsy or subtotal resection, female sex, higher performance status, application of chemoradiation, and MGMT status among others have been suggested as prognostic for OS in multiple studies [23–25]. To control for these factors, we chose a matched-pair design with one LTS group matched to two STS groups; unfortunately, we could only identify 80 matches for the 2nd (control) STS group which was therefore matched with an 80-patient fraction of the LTS group.

Using this approach, we observed an increased frequency of *IDH1* R132H mutations in the LTS group compared to STS-1. The difference was not significant in the second comparison with STS-2; therefore, we could not explicitly confirm the previously described prognostic significance of *IDH1* mutational status [26] in our dataset. The literature is inconsistent on the relevance of the tumor growth fraction in GB [12–15]; samples of patients in the LTS group had lower growth fraction indices compared to STS-1; however, despite a consistent numerical trend, the difference was not significant in the control analysis between LTS and STS-2.

In contrast, we corroborated the association of *MGMT* promoter hypermethylation [23–25] in our dataset as the LTS group contained a significantly increased frequency of methylated tumors compared to both STS-1, and STS-2. The frequency of *GFAP*-positive tumors was also significantly increased in the LTS group compared to both STS groups. Interestingly, both factors were not associated with each other and positivity for both was associated with the best, while negativity for both with the worst OS outcomes in our cohort, indicating an association of *GFAP* with OS which occurs independently of *MGMT* promoter hypermethylation.

The association of *GFAP* with OS is consistent with the loss of *GFAP* in high-grade tumors [18, 19] and increased tumor growth [20]; however, more recently, the relevance of different isoforms of *GFAP* [27] and an upregulation of *GFAP* in non-neoplastic astrocytes as a reaction to tumor growth have also been described by different groups [28, 29].

Our study has several limitations such as its retrospective nature which should be replicated in a prospective design; furthermore, a small proportion of LTS patients remained without a 2nd match, possibly impairing statistical power of the analysis. A preferrable approach would have included using a control dataset for both LTS and STS; however, we could not identify a sufficient number of patients for this end. Furthermore, the 2021 WHO classification of brain tumors updated the classification of glioblastoma to exclude IDH-mutant tumors [30]. Our study was conducted before this classification was implemented and all groups include patients with tumors classified as IDH-mutant glioblastoma which would now be classified as IDH-mutant astrocytoma (grade 4). For our study, we used histopathological data from routine clinical analysis; therefore, some panels or molecular data were missing. Finally, there was no central image review and no central histopathology review for the study; however, all patients were treated within the framework of an interdisciplinary tumor board setting which included imaging and histopathology reviews.

Despite these shortcomings, our study provides evidence for a prognostic role of GFAP positivity in GB tissue and further corroborates MGMT methylation as a prognostic marker for OS.

## Conclusion

We observed a significant association of MGMT promoter hypermethylation and GFAP expression with LTS in this cohort of GB patients. Given the hypothesis-generating nature of our study, these observations should be confirmed in prospective clinical trials.

## Supplementary Information

**Additional file 1**: **figure S1**. a) Overall survival in long-term survivors compared to short-term survivors-1. b) Overall survival in long-term survivors compared to short-term survivors-2.

## Data Availability

The dataset generated and analyzed in the current study are available from the corresponding author on reasonable request.

## Abbreviations

GB: Glioblastoma
GFAP: Glial fibrillary acidic protein
IDH-1: Isocitrate dehydrogenase 1
KPS: Karnofsky-performance-status
LTS: Long-term survivor
MGMT: O6-methylguanine-DNA methyltransferase
MIB-1: Molecular immunology Borstel-1
OS: Overall survival
PFS: Progression-free survival
STS: Short-term survivor
WHO: World Health Organization
