# Supplementary figures and images for: Molecular features of glioblastomas in long-term survivors compared to short-term survivors—a matched-pair analysis
Source: Radiat Oncol. 2022 Jan 24;17:15. doi: 10.1186/s13014-022-01984-w (PMC8785532; doi:10.1186/s13014-022-01984-w)

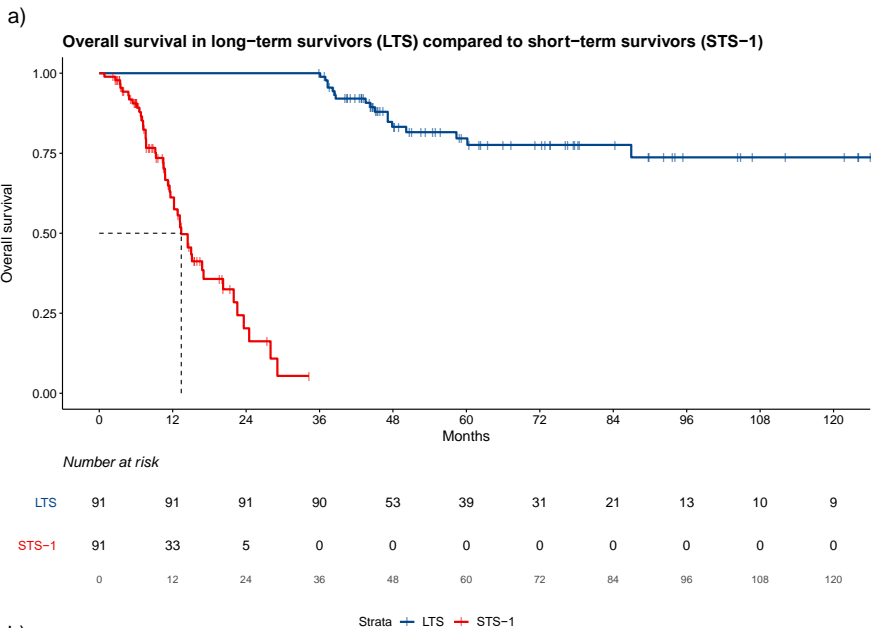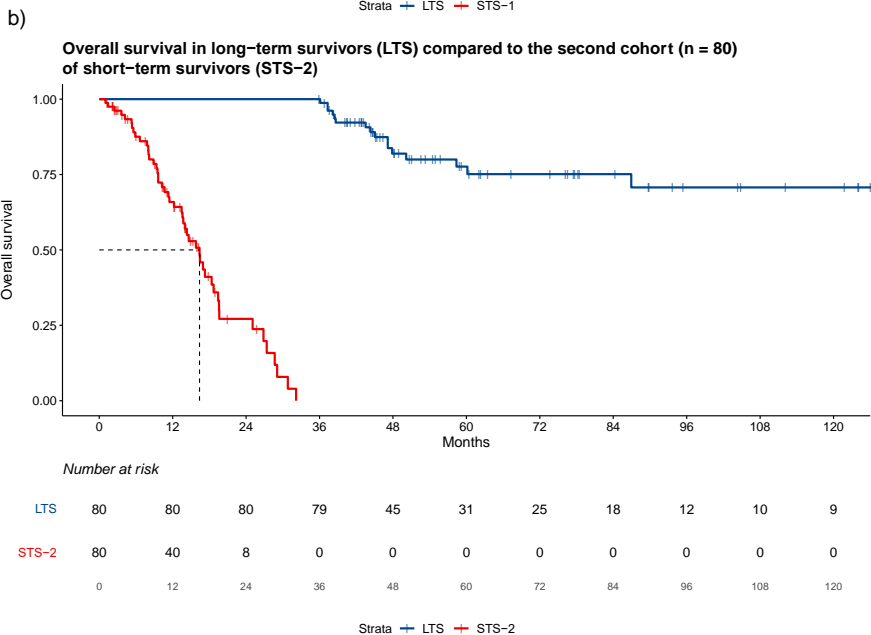

**Supplementary figure 1.**

Supplement: Supplementary file 1 — Additional file 1: figure S1. a) Overall survival in long-term survivors compared to short-term survivors-1. b) Overall survival in long-term survivors compared to short-term survivors-2. [file 13014_2022_1984_MOESM1_ESM.pdf]
